# Successful Case of Cæsarian Section

**Published:** 1853-07

**Authors:** 


*Successful Case of Cæsarian Section.*—Dr. Decoene relates the following case of a female, aged 30, upon whom he had once before performed the Cæsarian operation. He saw the patient in the seventh month of pregnancy, November 5, 1852, together with Dr. Frédérics. She had then been forty hours in labor; and stated that, on the previous evening, she had experienced a breaking or yielding sensation in the lower part of the abdomen, followed by discharges of blood from the vagina, insensibility, and sickness. Since that time, both labor-pains and the movements of the child had ceased. The whole abdomen was very tender. Examination *per vaginam* detected a relaxed os uteri, about the size of a dollar; but neither the head nor other part of the child could be felt, although the limbs could be traced through the abdominal walls. Inasmuch as the author concluded that the uterus had given way, and the child had escaped into the abdomen, he determined upon an operation. After making the preliminary incisions, and opening the peritoneum, he came upon a mass of congealed blood, under which was a male infant, with the separated placenta, and the membranes still entire. It was easily removed; then, to the operator's great astonishment, a second child, female, came to view. Both infants were dead. The uterus, which was contracted, exhibited along its front surface a slit, answering to the line of incision of the former operation. No hemorrhage ensued; the abdominal wound was united; and, by November 24, the patient was well.

Sichel observes, in reviewing the above in Schmidt's *Jahrbuch*, that women, who have the good fortune to recover after the performance of the Cæsarian section, appear to be singularly liable to rupture of the uterus along the line of the former incision in every subsequent pregnancy. Kaysen relates six such cases. The giving way of the cicatrix in the earlier months of pregnancy may lead to escape of the ovum, and the development of an extra-uterine growth.—*Med. Times and Gaz.*, May 21, 1853

